# The Use of Cutting Balloons in Published Cases of Acute Coronary Syndrome Caused by Spontaneous Coronary Artery Dissection

**DOI:** 10.31083/j.rcm2408235

**Published:** 2023-08-17

**Authors:** Svetlana Apostolovic, Bojan Maricic, Nenad Bozinovic, Tomislav Kostic, Zoran Perisic, Aleksandra Djokovic, Mihajlo Bojanovic, Milovan Petrovic, Goran Stankovic

**Affiliations:** ^1^Department of Cardiology, Division of Interventional Cardiology, University Clinical Center Nis, 18000 Nis, Serbia; ^2^Faculty of Medicine, Department of Internal Medicine, University of Nis, 18000 Nis, Serbia; ^3^Department of Cardiology, Division of Interventional Cardiology, University Hospital Center Bezanijska kosa, 11080 Belgrade, Serbia; ^4^Faculty of Medicine, Department of Internal Medicine, University of Belgrade, 11000 Belgrade, Serbia; ^5^Department of Cardiology, Division of Interventional Cardiology, Cardiovascular Institute Vojvodina, 21204 Sremska Kamenica, Serbia; ^6^Faculty of Medicine, Department of Internal Medicine, University of Novi Sad, 21000 Novi Sad, Serbia; ^7^Department of Cardiology, Division of Interventional Cardiology, University Clinical Center of Serbia, 11000 Belgrade, Serbia

**Keywords:** spontaneous coronary artery dissection, cutting balloon, acute coronary syndrome

## Abstract

Spontaneous coronary artery dissection (SCAD) is a non-traumatic, non-atherosclerotic layering of the coronary artery wall due to the presence of a subintimal hematoma or an intimal tear with the creation of a false lumen that compresses the true lumen and restricts or obstructs the flow. Patients with SCAD and preserved coronary flow are treated conservatively according to the general recommendations. However, percutaneous coronary intervention should be considered in patients with artery occlusion and/or refractory ischemia. Stenting is associated with increased risks comprising stenting in the false lumen, in-stent thrombosis, and/or stent malappositon as well as antegrade or retrograde propagation of the intramural hematoma. Intracoronary imaging is of great value both for the diagnosis and treatment of SCAD. There is rising scrutiny on the use of cutting balloons in acute coronary syndrome caused by SCAD. The idea of using cutting balloons is to fenestrate the intima and drain the intramural hematoma. Our review presents an analysis of 17 published cases of cutting balloon (CB) use in SCAD. What is encouraging is that of the 12 published cases, in 11 Thrombolysis in Myocardial Infarction (TIMI) 3 flow was established with this technique, and TIMI 2 flow in one, without subsequent stent implantation. Four patients received a stent after the CB use, while one patient underwent CB angioplasty after hematoma propagation caused by stent implantation. In all cases, patients were asymptomatic at follow-up, with TIMI 3 flow.

## 1. Introduction

Spontaneous coronary artery dissection (SCAD) is a non-traumatic, non-atherosclerotic layering of the coronary artery wall due to the presence of a subintimal hematoma or an intimal tear with the creation of a false lumen that compresses the true lumen and restricts or obstructs the flow [1].

Spontaneous coronary artery dissection can be classified based on angiographic findings as [2]:

• Type 1 (an obvious stain on the wall of the artery with the presence of a double lumen);

• Type 2 (diffuse smooth stenosis of varying degrees, usually >20–30 mm);

• Type 3 (focal or tubular stenosis mimicking atherosclerosis usually 11–20 mm);

• Type 4 (dissection leading to a sudden total occlusion, usually of the distal coronary segment).

The treatment of patients with SCAD still remains controversial and based on published clinical practice registries. Cutting balloon (CB) technique relying on the contemporary knowledge of SCAD pathophysiology, represents a potentially the least harmful therapeutic option bearing in mind that potent antiplatelet drugs and anticoagulants by preventing vessel healing and false lumen thrombosis, can facilitate disease progression and prolongation [3, 4, 5, 6, 7, 8, 9, 10, 11, 12, 13, 14, 15, 16, 17].

## 2. Materials and Methods

A systematic literature review was performed on three databases (EMBASE, Pubmed, Web of Science), from inception to 01/06/22. We used the following MeSH terms: SCAD, acute coronary syndrome, and cutting balloon. Papers in English/Serbo-Croatian, including SCAD patients treated with CB, were included. Non-human studies, pediatric age (<18 years), articles in a language other than those listed above, full-text not available, and literature reviews represented the main exclusion criteria. The final analysis included 17 cases of SCAD published between 2014 and 2022, treated with CB angioplasty. A single author (MB) performed the de-duplication using the reference software EndNote (version 20, Clarivate Analytics, Philadelphia, PA, USA).

Data were presented in terms of absolute frequencies (percentage), and mean ± standard deviation, according to the terms used in the manuscript of origin.

## 3. Results

Include Out of these, 16 patients were female and 1 male, with an average age of 46.9 ± 9.4 years. Fourteen patients presented with ST-elevation infarction (STEMI). 55.6% of patients had no risk factors for coronary disease (Table 1, Ref. [18, 19, 20, 21, 22, 23, 24, 25, 26, 27, 28, 29, 30, 31]). In 14 out of 17 patients (84%), a lesion was found on the left anterior descending (LAD) artery, with the medial segment involved in 88% of cases. In 16 patients, SCAD was primarily treated with CB, but in 1 case, CB was used after stenting and hematoma propagation [18, 19, 20, 21, 22, 23, 24, 25, 26, 27, 28, 29, 30, 31].

**Table 1.** **An analysis of the demographics, risk factors and clinical presentation of published cases**.

Author	Year of publication	Age	Sex	Presentation	History of CVD	Risk factors
Main *et al*. [18]	2018	62	F	STEMI	No	No
Yumoto *et al*. [19]	2014	47	F	STEMI	No	No
McGrath *et al*. [20]	2018	52	F	NSTEMI	No	No
Zghouzi *et al*. [21]	2021	72	F	STEMI	Yes	N/A
Sharma *et al*. [22]	2019	53	F	UAP	Yes	N/A
Kaya *et al*. [23]	2019	46	F	STEMI	No	No
Matsuura *et al*. [24]	2021	31	F	STEMI	No	Smoking
Alkhouli *et al*. [25]	2015	50	F	NSTEMI-STEMI	Yes	N/A
Ito *et al*. [26]	2016	46	F	STEMI	No	No
Bresson *et al*. [27]	2019	36	F	NSTEMI-STEMI	N/A	Smoking, oral contraceptives
Lee *et al*. [28]	2017	42	F	STEMI	No	N/A
Lee *et al*. [28]	2017	46	F	STEMI	No	N/A
Uema *et al*. [29]	2013	42	F	UAP-STEMI	Yes	Hyperlipidemia
Noguchi *et al*. [30]	2018	42	M	UAP-STEMI	No	Smoking
Bastante *et al*. [31]	2022	41	F	UAP	N/A	N/A
Bastante *et al*. [31]	2022	46	F	NSTEMI-STEMI	N/A	N/A
Bastante *et al*. [31]	2022	43	F	STEMI	N/A	N/A

F, female; M, male; STEMI, ST-elevation myocardial infarction; NSTEMI, Non-ST segment myocardial infarction; UAP, unstable angina pectoris; CVD, cardiovascular disease; N/A, not available.

In 9 out of 14 cases, a Boston Scientific Flextome© CB was used, while in 3 cases the name of the balloon was not specified. The diameter of the balloon ranged from 2 to 4 mm, with a length of 8 to 20 mm (Table 2, Ref. [18, 19, 20, 21, 22, 23, 24, 25, 26, 27, 28, 29, 30, 31]).

**Table 2.** **An analysis of the type and dimensions of CBs used in published cases**.

Author	Balloon type	Balloon diameter (mm)	Balloon length (mm)
Main *et al*. [18]	Boston Scientific Flextome ©	2.5	10
Yumoto *et al*. [19]	N/A	2.5	N/A
McGrath *et al*. [20]	Boston Scientific Flextome ©	3.0	10
Zghouzi *et al*. [21]	Boston Scientific ©	3.0	20
Sharma *et al*. [22]	Boston Scientific Flextome ©	4.0	10
Kaya *et al*. [23]	Boston Scientific Flextome ©	2.5	10
Matsuura *et al*. [24]	Boston Scientific Wolverine ©	3.5; 2.5	N/A
Alkhouli *et al*. [25]	Boston Scientific Flextome ©	2.0	10
Ito *et al*. [26]	Boston Scientific Flextome ©	2	N/A
Bresson *et al*. [27]	Boston Scientific Flextome ©	2.5	10
Lee *et al*. [28]	N/A	2.25	10
Lee *et al*. [28]	N/A	2.5	N/A
Uema *et al*. [29]	Boston Scientific Flextome ©	3	N/A
Noguchi *et al*. [30]	Boston Scientific Flextome ©	3 (LCx); 3.5 (LAD)	N/A
Bastante *et al*. [31]	Spectranetics AngioSculpt ©	2.5	15
Bastante *et al*. [31]	Spectranetics AngioSculpt ©	2.5	8
Bastante *et al*. [31]	Spectranetics AngioSculpt ©	2	20

CBs, cutting balloons; LAD, left anterior descending artery; LCx, left circumflex artery; N/A, not available.

Out of a total of 16 lesions affecting the medial segment (88%), 7 (44%) were limited only to the medial segment, while in 6 (38%) patients the lesions also included the distal segment, with a similar distribution for the apical, proximal and both apical and proximal segment (one patient). In 9 out of 12 (75%) cases, primarily treated with CB, the balloons were expanded in the medial segment of the affected artery, with repeat insufflation in 81% of cases.

In 69% of cases, the balloons were expanded in the middle of the dissection, and in 31% of cases in the distal part. They were most often expanded twice (50%) up to 2–4 atmospheres (ATMs) in 50% of cases, up to 6 ATMs in 37.5% of cases, and up to 8 ATMs in 12.5% of cases (Table 3, Ref. [18, 19, 20, 21, 22, 23, 24, 25, 26, 27, 28, 29, 30, 31]).

**Table 3.** **An analysis of the affected arteries as well as CB inflation details**.

Author	Balloon ATM	Number of inflations	Artery	Location	Inflation location
Main *et al*. [18]	3	2	DG1	Mid	Mid DG1
Yumoto *et al*. [19]	2–4	2	LAD	Mid - distal	Mid and dist LAD
McGrath *et al*. [20]	6	1	LM - LCx - OM1	Proximal	Proximal OM1 and proximal LCx
Zghouzi *et al*. [21]	/	2	LAD	Mid	Mid LAD
Sharma *et al*. [22]	6	3	RCA	Mid	Mid RCA
Kaya *et al*. [23]	4	3	LAD	Mid - distal	Mid and dist LAD
Matsuura *et al*. [24]	/	2	LAD	Mid - distal	Dist LAD
Alkhouli *et al*. [25]	2–4	2	LAD	Mid - apical	Mid and dist LAD
Ito *et al*. [26]	8	1	LAD	Mid - distal	Dist LAD
Bresson *et al*. [27]	/	/	LAD	Proximal - Mid	Mid LAD
Lee *et al*. [28]	/	2	LAD	Mid	Mid LAD
Lee *et al*. [28]	/	/	LAD	Mid - distal	/
Uema *et al*. [29]	/	/	LAD	Mid - distal	/
Noguchi *et al*. [30]	6	8 (LCx); 10 (LAD)	LM - LAD - LCx	LM = distal; LAD = Proximal - Mid; LCx = Proximal - Mid	Mid LCx; Proximal and mid LAD
Bastante *et al*. [31]	/	/	LAD	Mid	/
Bastante *et al*. [31]	/	/	LAD	Mid	/
Bastante *et al*. [31]	/	/	LAD	Mid	/

CB, cutting balloon; ATM, atmosphere; DG, diagonal branch; LAD, left anterior descending artery; LM, left main; OM, Obtuse marginal branch; LCx, left circumflex artery; RCA, right coronary artery; Mid, middle; Dist, distal.

In 70.6% of cases, the lesion was treated only with a CB, while in 29.4% of cases, a stent was implanted after balloon insufflation.

Intravascular imaging was used in 82% of cases. The representation of intravascular ultrasound (IVUS) compared to optical coherent tomography (OCT) was equal.

Out of 12 cases of isolated CB use, Thrombolysis in Myocardial Infarction (TIMI) 3 flow was established in 11 of them, while in one, TIMI 2 was the final result [18, 19, 21, 22, 23, 24, 28, 29, 31].

The most common imaging method used for follow-up was coronary angiography (CAG) (50%), performed over the time period of 13 days up to 6 months. In 25% of cases, computed tomography angiography (CTA) of the coronary arteries was performed over the period of 4 weeks to a year. An echocardiogram (EHO) was performed in 17% of cases, on discharge or after 6 weeks. In one case (8%), CAG and CTA were performed in tandem, CAG after 3 days, while CTA after one month and one year. Of all patients who underwent follow-up, complete healing of the dissection was observed in 30% of cases, while a residual intimal tear was present in 70% of cases (Table 4, Ref. [18, 19, 20, 21, 22, 23, 24, 25, 26, 27, 28, 29, 30, 31]).

**Table 4.** **An analysis of the follow-up imaging**.

Author	Treatment	Imaging follow-up	Follow-up time period	Imaging finding
Main *et al*. [18]	Cutting balloon angioplasty	EHO	6 weeks	LVEF to 60%, no regional wall motion abnormality
Yumoto *et al*. [19]	Cutting balloon angioplasty	CAG + OCT	6 months	residual partial intimal tear
McGrath *et al*. [20]	Cutting balloon angioplasty and stenting	/	/	/
Zghouzi *et al*. [21]	Cutting balloon angioplasty	CTA	4 weeks	healing of coronary artery dissection
Sharma *et al*. [22]	Cutting balloon angioplasty	/	/	/
Kaya *et al*. [23]	Cutting balloon angioplasty	CAG + CTA	CAG = 3 days; CTA = 1 month and 1 year	healing of coronary artery dissection
Matsuura *et al*. [24]	Cutting balloon angioplasty	CTA	10 months	healing of coronary artery dissection
Alkhouli *et al*. [25]	Cutting balloon angioplasty and stenting	/	/	/
Ito *et al*. [26]	Cutting balloon angioplasty	CTA	3 months	residual partial intimal tear
Bresson *et al*. [27]	Cutting balloon angioplasty and stenting	/	/	/
Lee *et al*. [28]	Cutting balloon angioplasty	/	/	/
Lee *et al*. [28]	Cutting balloon angioplasty and stenting	EHO	Upon discharge	LVEF 25–30% with severe hypokinesis
Uema *et al*. [29]	Cutting balloon angioplasty	CAG	13 days	residual partial intimal tear
Noguchi *et al*. [30]	Cutting balloon angioplasty and stenting	CAG	6 months	residual partial intimal tear
Bastante *et al*. [31]	Cutting balloon angioplasty	CAG	6 months	residual partial intimal tear
Bastante *et al*. [31]	Cutting balloon angioplasty	CAG	6 months	residual partial intimal tear
Bastante *et al*. [31]	Cutting balloon angioplasty	CAG	6 months	residual partial intimal tear

EHO, echocardiogram; CAG, coronary angiography; CTA, computed tomography angiography; LVEF, left ventricular ejection fraction; OCT, optical coherent tomography.

Patients in all cases were symptom-free at follow-up (Table 4).

## 4. Discussion

The treatment of patients with SCAD presenting as acute coronary syndrome (ACS) remains challenging. Most patients are treated according to general recommendations for patients with ACS, using a conservative antithrombotic/antiplatelet strategy. Potent antiplatelet drugs and anticoagulants are a double-edged sword because they can prevent progressive thrombus formation and vessel occlusion, but also by preventing vessel healing and false lumen thrombosis, they can also facilitate disease progression and prolongation [3, 4, 5, 6, 13, 32].

In our cases analyzed, LAD was the most common culprit artery in the ACS SCAD setting. Recent studies also reported that the LAD artery was the most commonly affected coronary artery in SCAD, with the middle and distal segments as the most common lesion site [8, 9].

**Fig. 1.**
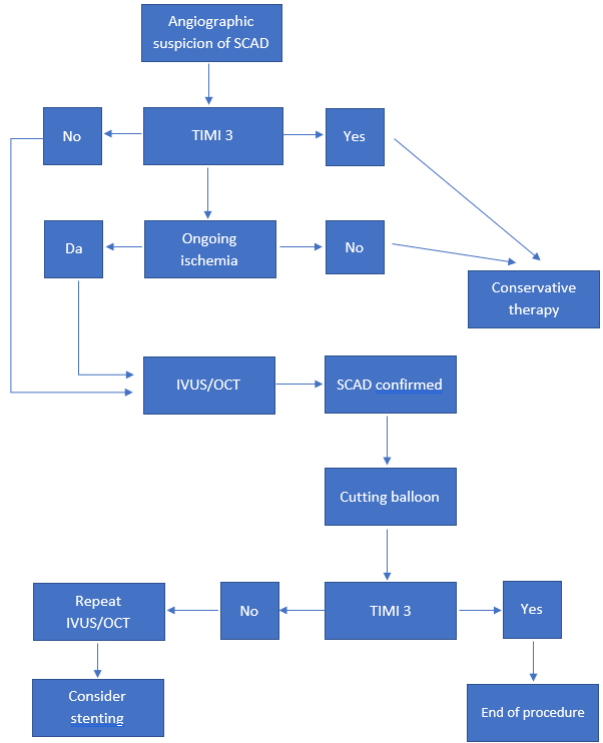
**An algorithm of suggested treatment procedure for SCAD**. SCAD, spontaneous coronary artery dissection; IVUS, intravascular ultrasound; OCT, optical coherent tomography; TIMI 3, Thrombolysis In Myocardial Infarction 3.

Percutaneous coronary intervention (PCI) revascularization should be considered in patients with arterial occlusion, ongoing or refractory ischemia [1, 7, 8, 9, 10, 11, 12] as well as SCAD of the main stem of the LCA (Fig. 1). PCI in this complex anatomical substrate is associated with technical difficulties, including difficult placement of the wire in the true lumen and the risk of dissection expansion, axial propagation of intramural hematoma, or side branch occlusion [13, 32].

**Fig. 2.**
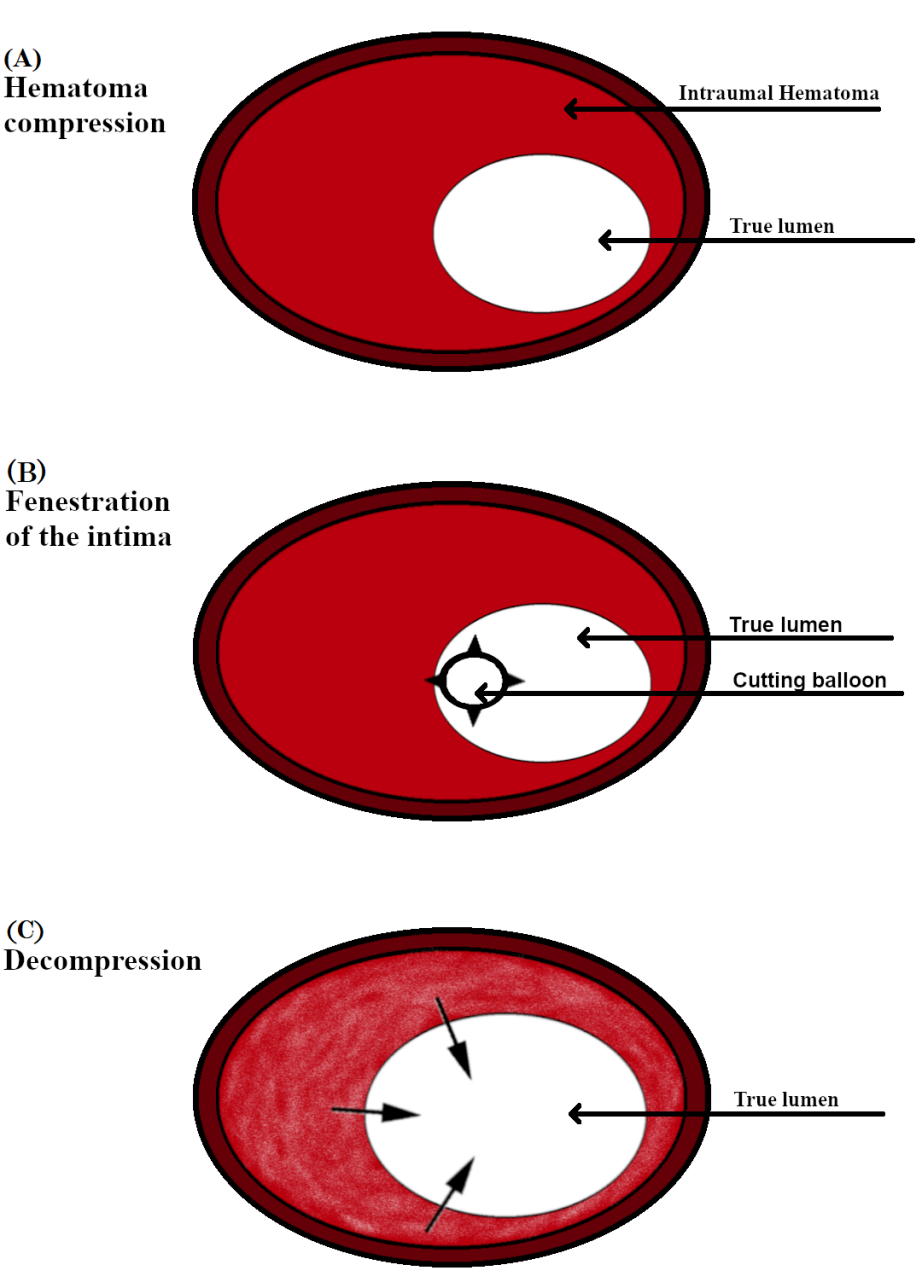
**A schematic presentation of CB application. **(A) The subintimal hematoma compresses the true lumen restricting blood flow. (B) Cutting balloon deployed on the site of the subintimal hematoma causing multiple fenestrations along the intima. (C) The fenestrations allow the blood to flow out from the false lumen tehereby decompressing the true lumen and improving blood flow. CB, cutting balloon.

Stenting is associated with significant risks such as stenting into a false lumen, in-stent thrombosis, and antegrade or retrograde propagation of intramural hematoma, especially when there is no existing intimal disruption. OCT studies showed that most cases of SCAD involve intramural hematoma without intimal dissection [32]. Fenestration of the intima with the CB creates communication between the false and true lumen, enabling decompression of the intramural hematoma, thus avoiding the need for stenting (Fig. 2). Intracoronary imaging is of great value for the diagnosis of SCAD. It is also of great importance for the guidance of angioplasty, by allowing: (a) correct positioning of the wire in the right lumen, (b) efficient inflation of the CB, with multiple entry sites, and (c) absence of insufficient expansion of the stent [25, 26, 27, 30].

In most published cases, if there was a resolution of ST dynamics and symptoms after the use of CB, stenting was not performed. There is good evidence that most SCADs will initially stabilize and then completely heal over time if treated conservatively (Fig. 1) [4, 13, 15, 16]. Revascularization in patients with SCAD is very challenging due to the presence of a disrupted and fragile coronary vessel wall. Compared with atherosclerotic myocardial infarction, the outcomes of PCI in SCAD are less predictable with higher complication rates and suboptimal outcomes [15, 33, 34]. Hematoma propagation occurs in up to one-third of PCI cases, often requiring the use of multiple unplanned stents [15]. PCI should be considered in ACS caused by SCAD when there is an artery occlusion or when the flow is compromised to such a degree that there is ongoing ischemia. Given the increased risk of adverse outcomes with PCI in SCAD, a number of less conventional interventional approaches have been proposed such as:

• Balloon angioplasty using small balloons with low pressure, followed by conservative therapy [35];

• Covering the proximal and distal ends of the affected segments with short stents in order to limit the hematoma before stenting the middle segment (“sandwich” technique) [36];

• Targeting the intimal tear or “flap” for focal stenting or stenting only the proximal extent of the dissection in order to prevent proximal propagation [33, 37];

• CB angioplasty in order to fenestrate the intimal and medial membrane and reduce pressure in the false lumen as an independent strategy or prior to stenting;

• Use of “bioabsorbable – scaffold” stents [38].

American College of Cardiology (ACC) state that coronary artery bypass grafting (CABG) is usually engaged in situations where PCI has failed or is considered of high risk (e.g., LM dissections with ongoing ischemia/infarction) [39].

So far, it has been shown that CB dimensions should be smaller than those of the blood vessels (mostly 2.5 mm used) and that insufflation should be performed at lower pressures (4–6 ATMs).

Several questions need to be addressed regarding the correct positioning of CB during angioplasty of the dissected segment:

(1) Should CB be used at the proximal segment with the idea of distal drainage into the artery?

(2) Is the correct position of the CB at the highest hematoma pressure and lumen narrowing (seen by intracoronary imaging method)?

(3) Or, is it best to use the balloon in two to three places in the artery?

What is encouraging is that out of the 12 published cases of isolated CB use in SCAD ACS, TIMI, 3 flow was established in 11 with this technique [18, 19, 21, 22, 23, 24, 26, 28, 29, 31]. Also, if a CB was used in the segment of hematoma propagation after stent implantation, the flow was restored [20].

## 5. Conclusions

The CB use in ACS caused by SCAD represents potentially the least harmful treatment option. Its true validation needs to be broadened from experiences based on several case reports with inevitable research bias to randomized studies. However, future randomized studies for CB angioplasty in ACS SCAD are challenging, namely for a proper methodology and study design which leave us to the individual treatment approach with the lowest level of possible harmful effects.
